# XII AIST 2018 Conference: “The thousand faces of cough: clinical and therapeutic updates”

**DOI:** 10.1186/s40248-018-0130-y

**Published:** 2018-07-02

**Authors:** Alessandro Zanasi, Antonio M. Morselli-Labate, Massimiliano Mazzolini, Marianna Mastroroberto, Roberto W. Dal Negro, Ivan Poliacek, Alyn H. Morice, Sara Maio, Giovanni Viegi, Jamie Koufman, Francesco Torresan, Alexandros Ioannou, Daniele Mandolesi, Elisa Liverani, Amedeo Montale, Franco Bazzoli, Fabio Baldi, Maurizio Zompatori, Giovanni A. Fontana, Ahmad Kantar, Peter Dicpinigaitis, Clive Page, Surinder S. Birring, Francesco Tursi

**Affiliations:** 1 Italian Association for Cough Study (AIST), Bologna, Italy; 2 Department of Medical and Surgical Sciences, University of Bologna Alma Mater Studiorum, Bologna, Italy; 3 National Centre for Respiratory Pharmacoeconomics and Pharmcoepidemiology, CESFAR, Verona, Italy; 4 Comenius University in Bratislava, Jessenius Faculty of Medicine in Martin, Medical Biophysics, Mala Hora 4, 03601 Martin, Slovakia; 5 Respiratory Medicine, University of Hull/Hull York Medical School, Castle Hill Hospital, Cottingham, East Yorkshire UK; 6 Institute of Clinical Physiology (IFC-CNR), Pisa, Italy; 7 Institute of Biomedicine and Molecular Immunology (IBIM-CNR), Palermo, Italy; 8 Voice Institute of New York, New York Eye and Ear Infirmary, Mt. Sinai Medical System, New York, USA; 9 Department of Medical and Surgical Sciences, Policlinico Sant’Orsola-Malpighi, University of Bologna, Bologna, Italy; 10 Centro di Studio e Ricerca sulle Malattie dell’Esofago - Università di Bologna, Bologna, Italy; 11 GVM Care & Research, VAH Bologna - MCH Cotignola, Cotignola, Ravenna Italy; 12 Bologna University, DIMES Dept, Bologna, Italy; 13 University of Florence, Florence, Italy; 14 Pediatric Asthma and Cough Centre, Istituti Ospedalieri Bergamaschi, University and Research Hospitals, Ponte San Pietro, BG Italy; 15 Albert Einstein College of Medicine and Montefiore Medical Center, Bronx, New York, USA; 16 Sackler Institute of Pulmonary Pharmacology, Institute Pharmaceutical Science, King’s College London, 150 Stamford Street, London, UK; 17 Respiratory Medicine, King’s College Hospital, London, UK; 18 Department of Pneumology, Lodi Hospital, Lodi, Italy

**Keywords:** Antitussive drugs, Cough, GERD, Pollution, Phenotypes, Respiratory diseases

## Abstract

This paper summarizes the presentations submitted for publication of the 12^th^ AIST National Congress (Associazione Italiana Studio Tosse/Italian Association for Cough Study) entitled “The thousand facets of cough. A clinical and therapeutic update”, which occurred last February 2^nd^-3^rd^, 2018 in Bologna (Italy). It summarizes the contributions from leading experts of the sector, who, as in the previous editions, also this year have analyzed a problem too often underestimated which still has many dark sides as regards both the diagnosis and the therapy of cough. The Scientific Committee has chosen topics that had less space in previous editions and these are topical subjects representing a concrete opportunity for learning and comparison of opinions, as well as indispensable elements for the correct management of the symptoms.

Hereby we report the abstracts of the works submitted for publication in this Meeting report.

The main topics have covered Cough relationship with nerve vagus, ATP, air pollution, GERD, imaging, COPD, pediatric and therapy. Of particular interest it is the preliminary data on cough hydration ratio that shows a highly significant correlation between dehydration and cough.

**Electronic supplementary material:**

The online version of this article (10.1186/s40248-018-0130-y) contains supplementary material, which is available to authorized users.

## Background

In the industrialized world cough is a growing medical, economic and social problem. The incidence of this symptom varies in the adult population from 5 to 40% based on environmental characteristics, season and habit of smoking. According to recent estimates of the SIMG (Italian Society of General Practice), acute cough involves 6 million specific consultations per year, and the chronic one involves over 3 million consultations: thus cough is the third cause that induces a patient to visit his doctor in Italy. In the pediatric field, a recent Italian epidemiological study, conducted in 2017 by Prof. Roberto Dal Negro, has found that over two thirds of Italian children present at least one cough episode during the year [1].

The XII Congress of the “Italian Association for the Study of Cough (AIST)” was held in Bologna at the S. Orsola Malpighi Hospital last 2nd and 3^rd^ of February 2018. Every 2 years since 1998, AIST meets the leading experts of the sector in Bologna, in order to give voice to a problem too often underestimated which still has many dark sides as regards to both the diagnosis and the therapy of cough. The 2018 edition program has only marginally touched important issues such as cough associated with asthma, rhinitis, auto-immune condition, pulmonary fibrosis.because they were widely discussed in the past editions and this meeting has proposed extremely topical subjects representing a concrete opportunity for learning and comparison of opinions, as well as indispensable elements for the correct management of the symptoms.

Hereby we report the abstracts of the works submitted in time for publication in this Meeting report.

## Main text

### Cough and hydration in children: Preliminary data (Alessandro Zanasi, Antonio M. Morselli-Labate, Massimiliano Mazzolini, Marianna Mastroroberto, Roberto W. Dal Negro)

Many studies have shown that children have a high tendency to dehydration, due to both educational problems and reduced sensitivity to thirst. Dehydration, in addition to neurological and renal problems, could lead to development or aggravation of respiratory diseases [2, 3].

Three hundred five children aged between 6 and 16 years selected from a free-living general population were evaluated for the presence of correlation between cough and dehydration through a data collection based on an anamnestic questionnaire investigating respiratory conditions and the collection of a sample of urine for the analysis of osmolality.

The results of the study show that only 39% of children drank enough water and the remaining 61% was dehydrated: 28% had moderate dehydration and 33% had severe dehydration. The questionnaire that investigated the prevalence of respiratory problems showed that 75% of children presented 1–2 cough episodes in the last year while only 25% reported that they never presented cough. As far as the presence of cough according to dehydration was concerned, 90% of dehydrated children presented cough during the last year versus 52% of those who were normo-hydrated (*p* = < 0.01. In particular, cough was present in 85.7 of children with moderate dehydration and 93.1 in those with severe dehydration.

In conclusion, the analysis of the data shows a highly significant correlation between dehydration and presence of cough: more the child is dehydrated and more he/she coughs.

### Vagus nerve and cough reflex (Ivan Poliacek)

The cough reflex fully depends on the activity conducted within the vagus nerve. Cough mechanosensors responding also to acid and sending their signal via A-delta fibers and non-specific primarily bronchial C-fiber cough chemosensors initiate cough. Cough related afferents terminate in the solitary tract nucleus.

We reexamined effects of unilateral vagal block on the tracheobronchial cough [4]. The cough number, expiratory and inspiratory motor output were markedly reduced by unilateral vagal cooling. Majority of temporal cough characteristics were prolonged. Unilateral vagotomy resulted in similar, however, less pronounced changes in coughing. It is mainly due to vagotomy executed on late adapted cough, which expresses characteristics similar to those during vagal cooling.

Among secondary cough afferent drives the role of volume feedback is controversial. We employed a modulation of lung volumes during cough by ventilator [5]. When tidal volume was delivered there was a significant increase in the inspiratory and the expiratory cough motor drive and the cough inspiratory phase duration. When the cough volume was delivered fast (within the half of the inspiratory period), the effects were opposite.

In conclusion, primary cough afferent drive arising from the stimulation of mechanosensitive cough receptors determine all three aspects of cough performance, the number of responses, the intensity of motor outputs and a temporal feature of cough motor pattern. Some cough modulation from secondary afferent feedback may become manifest under irregular, possibly pathological conditions.

### The role of ATP in cough (Alyn H. Morice)

ATP has emerged as a major mediator of cough hypersensitivity. The first evidence was provided by the trial by Abdulqawi et al. [6] performed in patients with chronic cough using the P2X3 (ATP) receptor blocker AF219/MK7264. A dramatic reduction in coughing over 24 h was recorded in this preliminary study. Subsequently, the potency of this drug has been shown by its effect at lower concentrations and an international Phase III study is about to be undertaken, which hopefully will provide us with the first novel antitussive medication in 40 years.

How blockade of the P2X3 receptors causes a reduction in cough hypersensitivity is currently unknown. We have conducted a study demonstrating that inhaled ATP causes a dose related cough [7] and that, unsurprisingly, MK7264 inhibited this inhalational challenge. Perhaps more interesting was that challenge with conventional protussive agents capsaicin and citric acid were unaffected by pre-administration of MK7264. A fourth challenge, distilled water fog, was diminished by MK7264 inferring that osmotic stimuli maybe regulated through P2X3. Work from Maria Belvisi’s laboratory has demonstrated that TRP4, an osmotically sensitive channel may ‘talk’ to the P2X3 and this TRP receptor may be the origin of hypersensitivity rather than the P2X3 receptor itself. Inhalation of ATP only causes a brief tussive response whereas the TRPV4 agonist produces prolonged depolarisation of afferent nerves. Clinical studies of TRPV4 antagonists are eagerly awaited.

### Air pollution and cough: an increasingly common combination (Sara Maio, Giovanni Viegi)

Air pollution is a well-established hazard to human health [8]. Air pollution is a major environmental health risk in both developing and developed countries. It has been estimated that globally 12.6 million deaths (23% of all deaths) are attributed to the environment [9].

Air pollution has both short-term and long-term adverse effects on respiratory health, for which cough is a main symptom. Short-term exposure, due to acute increase in air pollution, may cause premature mortality and increase hospital admissions for exacerbations of chronic obstructive pulmonary disease or asthma. Long-term cumulative health effects of chronic exposure comprise an increase in mortality and morbidity for respiratory diseases and impaired development of the lungs in children. In patients with chronic respiratory symptoms/diseases, continued exposure to noxious agents promotes a more rapid decline in lung function and increases the risk of repeated exacerbations [8].

In particular, as regards cough, a recent meta-analysis showed that fine particulate matter (PM_2.5_) was associated with significant higher risk of having cough (OR 1.05), especially in children (OR 1.08) [10]. Living near major roads was also associated with a higher prevalence of chronic cough, with the highest risk value in subjects living nearest the major road: OR 2.54 in subjects living within 100 m and OR 1.97 in subjects living between 100 m and 200 m (with respect to those living at a distance > 200 m) [11]. A recent Italian study showed an important increase in usual cough prevalence in a general population sample in the last 25 years (from 11.4 to 16.5%). Living in urban area resulted a borderline risk factor for usual cough (OR 1.14) [12].

Long-term exposure to ambient air pollution could cause cough in healthy girls [13]. These results were confirmed by a more recent study showing that living in urban area had the potential to trigger cough and to cause cough hypersensitivity, in particular in school-aged girls, suggesting an implication of several biological and behavioral factors, changing with age and gender, in the relation between air pollution and cough [14].

In conclusion, these recent scientific evidences showed a strong relationship between air pollution and cough in adults and in children.

### Respiratory reflux, the vagus, and chronic cough (Jamie Koufman)

Fragmentation of the aerodigestive tract into narrow, discrete, and anatomic medical specialties has resulted in disorganized and substandard healthcare for patients with chronic cough and most other respiratory conditions. Unfortunately, the diagnostic technology is archaic and inadequate; and generally medical professionals are not trained to recognize or manage respiratory reflux (aka LPR) or vagally-mediated neurogenic syndromes (Table 1) [15–22].

**Table 1:** Respiratory reflux and neurogenic syndromes

Respiratory reflux integrated aerodigestive medicine	
Chronic cough^†^	Asthma^†^
Chronic bronchitis	Allergy
Shortness of breath^†^	Inability to take a full breath^†^
Chronic obstructive lung disease	Recurrent pneumonia
Idiopathic pulmonary fibrosis	Snoring and Sleep apnea
Chronic throat clearing	Excessive nose & throat mucus
Post-nasal drip	Sinusitis
Paradoxical VF movement^†^	Laryngospasm^†^
Dysphonia^†^	Odynophonia^†^
Globus^†^	Dysphagia
Sore throat^†^	Throatburn^†^
Gastroparesis^†^	Odynophagia^†^
Big uvula syndrome	Ear blockage
Laryngeal & Airway stenosis	Aerodigestive tract cancer

^†^Often associated with neurogenic vagal dysfunction

The author presents her diagnostic paradigm as well as data from a series of fifty consecutive chronic cough patients (mean duration of cough 10 years). Work up consisted of: (1) Chest radiograph, PPD, and PFTs prior to being seen by the author; (2) comprehensive history including the glottal closure index (GCI), the reflux symptom index (RSI), and the Koufman chronic cough index (KCCI) (provided as an Additional file 2); (3) transnasal flexible laryngoscopy (TFL) with videostroboscopy and scoring of the reflux finding score (RFS); (4) pharyngeal/UES/esophageal manometry; (5) ambulatory ISFET double-probe (simultaneous pharyngeal and esophageal) pH-monitoring; and (6) laryngeal electromyography (LEMG) for those with VF paresis seen or suspected on TFL.

The subjects’ final diagnoses were: 40% respiratory reflux only, 14% neurogenic only, and 46% both. (There were no other diagnoses in the study group.) Out of the 26 patients with “asthma,” only 20% (5) actually had it. On transnasal esophagoscopy, 64% (32) had esophageal pathology.

Treatment for reflux-related and neurogenic cough consisted of dietary and lifestyle modifications, H2-antagonists, alginates, and when indicated, amitriptyline and/or gabapenten (that had to be titrated for each individual patient. The mean pre-treatment score— on a 0–5 cough severity scale—was 4.3; and post-treatment score was 1.1 (*p* < 0.0001). The post-treatment results summarized: no or minimal cough 78% (39); moderate cough 18% (9); and severe cough 4% (2).

Reflux-related and neurogenic cough are ubiquitous and dominate the “field” of chronic cough. Because contemporary diagnostics are inadequate, misdiagnosis and mismanagement are prevalent. This presentation offers a new approach, one that is based upon the presumption that vagal dysfunction and reflux are the root causes of chronic cough in most of our patients (see Additional files 1 and 2).

### Extraesophagel manifestations of gastroesophageal reflux disease (GERD) (Franco Torresan, Alexandros Ioannou, Daniela Mandolesi, Elisa Liverani, Amedeo Montale, Franco Bazzoli)

Extraesophageal manifestations of gastroesophageal reflux disease (GERD) include cough, laryngopharingeal reflux (LPR) and asthma and are the most frequent reasons of medical examination with an important economic burden (almost 50 billions dollars a year in the United States), of which 85% could be attribute to pharmacological costs.

LPR consists in reflux of gastric contents beyond the upper esophageal sphincter, triggering chronic laryngitis or laryngopharingitis. Chronic cough might be induced by gastric reflux, that causes irritation of the larynx and the activation of the afferent limb of the cough reflex. Asthma is typically caused by direct irritation of the tracheobronchial tree after aspiration of gastric contents into the airway, causing direct irritation and bronchoconstriction, or by stimulating the esophageal-bronchial neural reflex arc through the vagus nerve that leads to bronchoconstriction.

This disease besides gastric reflux could be also caused by a great variety of agents. Distinguishing whether cough, LPR, and asthma are caused by GERD remains challenging for both the primary care physician and the specialist. This distinction is important because treatment of GERD with the intent of improving or curing extraesophageal manifestations can be ineffective. Considering that patients with extraesophageal manifestations of GERD usually do not report typical GERD symptoms (heartburn and regurgitation), the probability to mistreat with proton pump inhibitors (PPI) patients without GERD is quite common.

Patients with chronic cough and typical symptoms of GERD or those whose chronic cough persists after ruling out upper airway disease, asthma or non-asthmatic eosinophilic bronchitis should undergo medical treatment for GERD, with dietary and lifestyle modifications, and acid suppression therapy. Response should be assessed 1 to 3 months after initial therapy.

Patients without typical symptoms of GERD and those of the previous group whose cough does not improve after antisecretory therapy should undergo esophageal MII-pH monitoring off-therapy in order to diagnose GERD and eventually repeated on therapy in non responders patients to detect the reason of antisecretory therapy failure.

### Cough: Real diagnostic and therapeutic value of PPIs (Fabio Baldi)

PPIs (Proton Pump Inhibitors) still represent a cornerstone of pharmacologic treatment for gastroesophageal reflux disease (GERD) because of their unquestionable efficacy in reducing gastric acidity. They are widely prescribed to patients who complain of symptoms ascribed to GERD including either typical manifestations such as heartburn, regurgitation or chest pain and extraesophageal manifestations such laryngitis, cough and asthma. However, literature data coming from controlled studies have clearly shown that the PPI efficacy in treating the clinical manifestations of GERD is rather variable ranging from a success rate of more than 75% in patients with esophagitis to far less than 50% in those with extraesophagel symptoms [23]. Therefore, when deciding to prescribe PPIs to a patient with chronic cough attributed to GERD we should be aware that our decision may not achieve the expected results. The factors that may lead to this unsatisfaction can be summarized in 3 main groups. The first is a poor diagnostic accuracy and consequently a bad patient selection. We must remember that in patients with chronic cough the probability of a successful PPI therapy strictly depends on the probability that an acid reflux is the cause of symptoms. It is important that this assumption is based on clinical features (i.e. Simultaneous presence of typical reflux symptoms) and on functional data (i.e. Reflux detection with pHmetry). Moreover, we can use PPIs as a diagnostic tool since it has been shown that an initial treatment with a double daily dose for 4 weeks may be helpful for selecting patients with cough who will subsequently benefit of a PPI therapy [24]. The second group of factors concerns the effect of PPI administration on GERD: these drugs have a very specific and selective action targeted to the inhibition of acid secretion by the gastric parietal cells. They cannot be considered as anti-reflux drug since they do not counteract the phenomenon of GERD. Studies performed with a methodology (pH-impedance) that allows the detection of reflux independently of its pH, have shown that during PPI administration the esophageal acid exposure is reduced but the total number of reflux is unchanged due to the persistence of reflux episodes with a low or absent acidity. Therefore, if we assume that the cough episodes may be triggered by the presence and proximal extension of refluxate independently of its pH we understand one of the reasons of PPI failure. The last group of factors that can explain the lack of response to the PPI administration is represented by the co-existence of other mechanisms involved in the pathogenesis of cough. The most important of them is probably the so-called “cough hypersensitivity syndrome” due to the sensitization of both the mucosal and central receptors. This is a difficult to treat condition and requires the use of neuroleptic drugs. In conclusion, PPIs are very effective and safe drugs but we must take into account their limitations when we choose them for the treatment of chronic cough attributed to GERD.

### Imaging and cough (Maurizio Zompatori)

In 5% of the adult patients with acute cough (AC) and fever chest X ray shows pneumonitis.

The diagnosis (dx) of pneumonitis requires a confirmation with radiological imaging.

However, AC due to infections related to atypical agents (ex; *Mycoplasma Pneumoniae*) can be associated with a normal or borderline chest X ray.

In these cases, HRCT can show bronchial wall thickenig, tree in bud or ground glass centrilobular opacities, small areas of consolidation.

Excluding pneumonitis, chest X ray has a limited role and can change the patient management only in a small percentage of the patients with AC (ex: detection of pneumothorax, pleural effusion or previously unsuspected intestitial pulmonary edema).

Chest X ray is also useful in the work up of a suspected pulmonary embolism but cannot establish the dx by itself and the angioCT study is usually required.

Chest X ray can also demonstrate complications of severe AC, such as rib fractures.

In case of chronic cough (CC) [25–28], imaging techniques can be instrumental in defining the etiology, for example post nasal drip (sinusal X ray, CT, MR), infections, bronchiectasis and a lot of more rare but not less important conditions, especially detectable using CT (tumors, TBC, interstitial lung diseases, chronic pulmonary embolism and pulmonary hypertension etc).

In conclusion, imaging appears to be generally more useful in chronic than in acute cough.

The diagnostic algorithms are not completely defined yet and the role of imaging techniques is not always clear, however chest X ray is advised in the existing guidelines for patients with chronic cough and no history of ACE-I therapy.

Chest X ray and sinusal imaging are often required before starting empiric treatments.

The indications for contrast enhanced CT and HRCT should be evaluated using a multidisciplined and tailored approach.

### Cough in COPD (Giovanni A. Fontana)

Until recently, chronic obstructive pulmonary disease (COPD) has been assessed based on airflow limitation, with little attention to the accompanying symptoms. It was only with the last issue of the GOLD guidelines [29] that the relevance of symptoms, namely chronic cough, in the natural history of the disease has been acknowledged. The reasons for this are likely due to the lack of knowledge on the association between cough and the clinical presentation and evolution of COPD [30]. In recent years, it has become progressively clear that COPD patients with chronic productive cough have a worse lung function and general health status, as well as an increased risk for exacerbation and death. Airway inflammation, excess airway mucus, continued inhalation of irritant cigarette smoke, along with co-morbidities such as bronchiectasis and gastro-oesophagel reflux, are thought to contribute in the chronic cough of COPD patients. At present we need a more systematic study of this important symptom in larger numbers of well characterised patients using validated ways of assessing the occurrence and impact of cough. Once we better understand the nature and variability of this symptom at a patient level, then we will be able to develop more effective methods of managing it in clinical practice.

### Cough phenotypes in children (Ahmad Kantar)

Despite the high prevalence of cough in children, the subject is relatively poorly researched. Although to pediatricians children are clearly different to adults, this seems less recognizable to many health professionals. During childhood, the respiratory tract and nervous system undergo a series of anatomical and physiological maturation processes that influence the cough reflex. In addition, immunological response undergoes developmental and memorial processes that make infection and congenital abnormalities the overwhelming causes of cough in children. The unavailability of comprehensive clinical data regarding chronic cough in children, has initially confined pediatrician to adopt an adult’s approach to the problem. Recent research had urged to a rethinking about the etiology of chronic cough in children. Attention is now focused on protacted bacterial bronchitis (PBB) and malacia as major causes of chronic cough in preschool age and as a possible precursor of bronchiectasis. New horizons of research are now emerging not only in treatment but also in prevention of some causes of chronic cough in children. Chronic cough in children may be representative of a simple, spontaneously resolving cough or a specific, serious disorder. Children with chronic cough should be evaluated carefully using protocols specific to children. Pediatric guidelines and clinical algorithms have identified pointers or “red flags” to consider during investigations. A correct interpretation of the phenotypic presentation can be translated into guidance for workup in primary care.

### Neuronal nicotinic receptor (NNR) agonists as potential antitussive drugs (Peter Dicpinigaitis)

Multiple studies have shown that cigarette smokers have suppressed cough reflex sensitivity compared with nonsmokers [31, 32].These observations implicated a component of tobacco smoke as the antitussive substance. Subsequent work evaluating the effect of electronic cigarette (e-cig) use in healthy volunteers demonstrated suppression of capsaicin-induced cough with nicotine containing, but not nicotine free e-cigs, thus implicating nicotine as the antitussive substance within tobacco smoke and e-cig vapor [33]. Experiments in guinea pigs demonstrated the antitussive effect of centrally injected nicotine [34], thus supporting the hypothesis generated in human studies. Although demonstrated to be antitussive, nicotine is not a viable therapeutic agent for the treatment of pathological cough given its numerous liabilities, including cardiovascular and gastrointestinal side effects, and risk of addiction.

There are many neuronal nicotinic receptor (NNR) subtypes. Animal studies to date have suggested that agonists of the alpha-_7_ NNR subtype account for most if not all of the antitussive effect of nicotine [35], and human clinical trials evaluating other patient populations have demonstrated alpha-_7_ agonists to be safe and well tolerated [36]. Thus, clinical trials are planned to assess the effect of a selective alpha-_7_ NNR agonist in patients with refractory chronic cough.

### Towards an improved preclinical model of hypertussive responses for the evaluation of anti-tussive drugs (Clive Page)

Cough remains a major unmet clinical need and current preclinical animal models have been poorly predictive for the activity of new antitussive agents in patients. We have established a novel method for inducing hypertussive responses in guinea pigs and rabbits following exposure to the pollutant gas ozone. We have observed a significant increase in cough frequency and a decrease in time to first cough to inhaled citric acid in both conscious guinea pigs and rabbits following exposure to ozone [37, 38]. This response was inhibited by the established antitussive drugs codeine and levodropropizine. In guinea pigs, hypertussive responses were also inhibited by bronchodilator drugs (β2 agonists or muscarinic receptor antagonists), although no such inhibition was seen in rabbits. These observations suggest that the observed hypertussive state in the rabbit was not secondary to bronchoconstriction. The ozone-induced hypertussive response in the rabbit was also inhibited by chronic pretreatment with capsaicin, suggestive of a role for sensitization of airway sensory nerve fibres. However, this hypertussive effects was not inhibited by TRPA1 antagonists, suggesting that ozone was not sensitizing airway sensory nerves via activation of this receptor. Whereas the ozone-induced hypertussive response was accompanied by a significant influx of neutrophils into the airway, the hypertussive response was not inhibited by the anti-inflammatory phosphodiesterase 4 inhibitor roflumilast at a dose that clearly exhibited anti-inflammatory activity. Furthermore, we have shown that carcaicinium chloride is effective in this model, a drug that has been recently been shown to inhibit cough in a pilot study in patients with chronic cough [37, 39]. In summary, our results suggest that ozone-induced hypertussive responses to citric acid may provide a useful model for the investigation of novel drugs for the treatment of hypertussive states, but some important differences were noted between rabbits and guinea-pigs with respect to sensitivity to bronchodilator drugs.

### Refractory chronic cough (Surinder S. Birring)

Refractory chronic cough (often referred to as idiopathic, unexplained or cough hypersensitivity syndrome) is a condition when cough persists despite the treatment of associated co-morbid conditions such as asthma, gastro oesophageal reflux and rhinitis [40] The clinical phenotype of patients is striking; they are predominantly female, onset in middle age, dry or minimally productive of sputum and the presence of laryngeal paraesthesia, such as a tickle sensation in the throat [41, 42]. Patients with refractory chronic cough have a hypersensitive cough reflex, and this can be demonstrated with cough challenge tests, using inhaled capsaicin or citric acid [43]. It is not known why patients develop refractory chronic cough, but possibilities include the persistence of a hypersensitive cough reflex following an upper respiratory tract infection, the menopause or the development of an auto-immune condition [44]. Recent studies have gained insights into the pathophysiology of refractory chronic cough. It is likely to be a disorder of dysfunctional cough sensory nerves. Peripheral and central sensitisation and reduced cough inhibition are likely to be important neural mechanisms [45].

There are evidence-based treatment options available that target central mechanisms. Neuromodulator drugs such as Gabapentin, widely used in neuropathic pain are also effective in refractory chronic cough [46]. Gabapentin requires dose titration at initiation to minimise side-effects such as drowsiness. Amitriptyline and Pregabalin are alternative options [47]. Speech or physiotherapy self-help interventions are also effective. In one multi-centre randomised controlled trial, physiotherapy and speech therapy intervention reduced cough frequency by 41%, compared to control. The effect size was comparable or better than some pharmacological alternatives [48]. A combination of neuromodulator drug and speech/physiotherapy has recently been reported to have superior and sustained anti-tussive efficacy compared to speech therapy alone [47]. A third option for treating patients with refractory chronic cough is opiate therapy. Low-dose Morphine has been reported to be effective and well tolerated in a randomised controlled trial [49].

There has been a rapid growth in research evaluating novel treatments for refractory chronic cough. Whilst some treatment targets have been disappointing in recent clinical trials, such TRPV1, TRPA1, selective sodium channels inhibitors and Lignocaine, there are many others that are promising. Clinical trials are being planned for 2018–2019 that will evaluate inhibitors of neural receptors P2X3, Neurokinin-1 and TRPV4. The most promising treatment in development is inhibitors of the P2X3 receptor such as MK7264 (AF219) [6]. It is very encouraging that refractory chronic cough is at long last being recognised as a disorder with a significant unmet clinical need, and there is much hope that developing treatments that target the neuronal mechanisms of cough will yield effective anti-tussive medications for patients.

### Efficacy and safety of a homeopathic syrup in URTI cough management: a review of two clinical trials in the pediatric and adult population (Alessandro Zanasi, Massimiliano Mazzolini, Antonio Maria Morselli-Labate)

With the aim of assessing the efficacy and safety of the homeopathic syrup containing Pulsatilla 6 CH, *Rumex crispus* 6 CH, Bryonia 3 CH, Ipeca 3 CH, Spongia tosta 3 CH, Sticta pulmonaria 3 CH, Antimonium tartaricum 6 CH, Myocarde 6 CH, Coccus cacti 3 CH and Drosera TM in the management of acute cough secondary to upper respiratory tract infections (URTI) in the pediatric and adult population, a review of two clinical studies was conducted: a real-life preliminary observational study in a pediatric population (*n* = 85) and a randomized, double-blind, placebo-controlled trial in an adult population (*n* = 80). In the pediatric study (*n* = 46 treated with homeopathic syrup; *n* = 39 treated with the addition of antibiotics), the verbal-category descriptive (VCD) score was significantly reduced (*p* < 0.001) and comparable in both groups starting from the second day of treatment, with complete cough resolution after 8 days in both groups. In the group treated with the homeopathic syrup, 4.3% reported adverse effects (AE) versus 23.1% in the group treated with the addition of antibiotics. In the adult study (*n* = 40 treated with homeopathic syrup; *n* = 40 treated with placebo), cough severity was significantly reduced in the group treated with the homeopathic syrup compared to the placebo (*p* < 0.001 vs. *p* = 0.023), reporting a greater reduction of sputum viscosity without any adverse effects related to treatment. This review demonstrates that the homeopathic syrup is effective in relieving URTI cough in children and adults, with no difference compared to the addition of antibiotics and more rapidly than the placebo, showing a higher safety profile compared to antibiotic medicines [50, 51].

### Acute cough in children: antibiotic towards symptomatic therapy (Alessandro Zanasi, Massimiliano Mazzolini, Francesco Tursi)

The use of antibiotic in URTI-related cough is strongly discussed and its routinary use is considered as a “malpractice”. Antitussive drugs are often not effective and sometimes they have undesirable effects especially in children [52, 53]. This is the reason that leads many people to approach natural medicine.

The goals of this study were:1) to verify the real utility of antibiotic therapy in reducing severity and duration of URTI related cough in children compared to symptomatic phytotherapeutic treatment (aerosol of *Pinus mugo* lavender essential oil, helichrysum and sink); and 2) to evaluate the safety of the treatments.

One hundred fifty nine children affected by URTI related cough were considered in this observational study. 52 children (group A) received only antibiotic for 7 days (amoxicillin or clarithromycin), 61 children (group B) received 2 aerosol/day with symptomatic phytotherapeutic agents for 7 days, and 56 children (group C) received aerosol therapy with symptomatic phytotherapeutic agents together with antibiotic therapy (amoxicillin or clarithromycin) for 1 week. Severity and duration of the cough were evaluated before and 7 and 15 days after treatment by using a VCD (verbal category descriptive) scale.

The results of the study show that the percentage of children in which the cough was resolved or improved after 7 days was significantly (*p* > 0.001) higher in groups B and C (respectively) when compared to group A.

In conclusion, our data confirm the poor efficacy of antibiotic treatment in children affected by URTI related cough, while they show the positive effect of aerosol therapy with balsamic (*Pinus mugo*, lavender, helichrysum and sink) agents in reducing the intensity and duration of the cough.

## Additional files

Additional file 1: Voice Institute of New York Chronic Cough Form. (DOCX 13 kb)

Additional file 2: Koufman Chronic Cough Index™ (KCCI) (*R = Reflux, N = Neurogenic*) *Ratio*. (DOCX 14 kb)

## Abbreviations

AC: Acute cough
COPD: Chronic obstructive pulmonary disease
GCI: Glottal closure index
GERD: Gastroesophageal reflux disease
KCCI: Koufman chronic cough index
LEMG: Laryngeal electromyography
LPR: Laryngopharingeal reflux
NNR: Neuronal Nicotinic Receptor
PBB: Protacted bacterial bronchitis
PPIs: Proton Pump Inhibitors
RFS: Reflux finding score
TFL: Transnasal flexible laryngoscopy
URTI: Upper respiratory tract infection
