# Supplementary material for: XII AIST 2018 Conference: “The thousand faces of cough: clinical and therapeutic updates”
Source: Multidiscip Respir Med. 2018 Jul 2;13:17. doi: 10.1186/s40248-018-0130-y (PMC6027558; doi:10.1186/s40248-018-0130-y)
Supplement: Supplementary file 1 — Voice Institute of New York Chronic Cough Form. (DOCX 13 kb) [file 40248_2018_130_MOESM1_ESM.docx]

**Additional file 1 Voice Institute of New York Chronic Cough Form**

Is your main problem COUGH? ______ ; For how many years? _____.

When your cough began, had you had a respiratory infection, cold, the flu, or other illness?____________________________________________________ .

Had a chest x-ray within the last two years? _______;Normal? ________.

Do you have a pulmonologist (lung doctor)? _______________________.

Are you on blood pressure medicine? _____; Which?_________________.
