# Supplementary material for: XII AIST 2018 Conference: “The thousand faces of cough: clinical and therapeutic updates”
Source: Multidiscip Respir Med. 2018 Jul 2;13:17. doi: 10.1186/s40248-018-0130-y (PMC6027558; doi:10.1186/s40248-018-0130-y)
Supplement: Supplementary file 2 — Koufman Chronic Cough Index™ (KCCI) (R = Reflux, N = Neurogenic) Ratio. (DOCX 14 kb) [file 40248_2018_130_MOESM2_ESM.docx]

**Additional file 2 Koufman Chronic Cough Index™ (KCCI) (*R= Reflux, N= Neurogenic*)**

*Please circle “Yes” or “No” for all ten questions (no maybes***)**

Do you awaken from a sound sleep coughing YES NO

violently, with or without trouble breathing?

Do you have choking episodes when you cannot YES NO

get enough air, gasping for air?

Do you usually cough when you lie down into YES NO

the bed, or when you just lie down to rest?

Do you usually cough after meals or eating? YES NO

Do you cough when (or after) you bend over? YES NO

Do you more-or-less cough all day long? NO YES

Does change of temperature make you cough? NO YES

Does laughing or chuckling cause you to cough? NO YES

Do fumes (perfume, automobile fumes, burned NO YES

toast, etc.) cause you to cough?

Does speaking, singing, or talking on the phone NO YES

cause you to cough?
  **R_______│_______N**

***Now, add the two columns up to derive the Reflux-to-Neurogenic ratio***
